# Discovery and evaluation of ZT55, a novel highly-selective tyrosine kinase inhibitor of JAK2^V617F^ against myeloproliferative neoplasms

**DOI:** 10.1186/s13046-019-1062-x

**Published:** 2019-02-04

**Authors:** Min Hu, Chengbo Xu, Chao Yang, Hongli Zuo, Chengjuan Chen, Dan Zhang, Gaona Shi, Wenjie Wang, Jiangong Shi, Tiantai Zhang

**Affiliations:** 1 State Key Laboratory of Bioactive Substance and Function of Natural Medicines, Institute of Materia Medica, Chinese Academy of Medical Sciences & Peking Union Medical College, Beijing, 100050 China; 2 Department of Blood Transfusion, General Hospital of the PLA Rocket Force, Beijing, 100088 China; 3 Department of Hematology, 307 Hospital of the PLA, Beijing, 100071 China; 4 Department of Pharmacy, China-Japan Friendship Hospital, Beijing, 100029 China; 5 School of Pharmacy, Collaborative Innovation Center of Advanced Drug Delivery System and Biotech Drugs in Universities of Shandong, Yantai University, Yantai, 264005 China

**Keywords:** JAK2^V617F^, JAK2 inhibitor, Myeloproliferative neoplasms, Apoptosis, ZT55

## Abstract

**Background:**

The JAK2-STAT signaling pathway plays a critical role in myeloproliferative neoplasms (MPN). An activating mutation in JAK2 (V617F) is present in ~ 95% of polycythemia vera, essential thrombocythemia, and primary myelofibrosis cases. This study aims to explore the selective JAK2^V617F^ inhibitor, evaluate the efficacy and possible mechanism of ZT55 on MPN.

**Methods:**

HTRF assays were conducted to evaluate the selective inhibition of ZT55 for JAKs. Cell apoptosis, proliferation, and cycle arrest assays were performed to examine the effect of ZT55 on HEL cell line with JAK2^V617F^ mutation in vitro. Western analysis was used to monitor the expression and activity of proteins on JAK2/STAT pathway. A mice xenograft model was established to evaluate the antitumor efficacy of ZT55 in vivo. Peripheral blood samples from patients with the JAK2^V617F^ mutation were collected to estimate the effect of ZT55 on erythroid colony formation by colony-forming assay.

**Results:**

We found that ZT55 showed a selective inhibition of a 0.031 μM IC_50_ value against JAK2. It exhibited potent effects on the cellular JAK-STAT pathway, inhibiting tyrosine phosphorylation in JAK2^V617F^ and downstream STAT3/5 transcription factors. ZT55 inhibited the proliferation of the JAK2^V617F^-expressing HEL cell line, leading to cell cycle arrest at the G_2_/M phase and induction of caspase-dependent apoptosis. Notably, ZT55 also significantly suppressed the growth of HEL xenograft tumors in vivo. Further evaluation indicated that ZT55 blocked erythroid colony formation of peripheral blood hematopoietic progenitors from patients carrying the JAK2^V617F^ mutation.

**Conclusion:**

These results suggest that ZT55 is a highly-selective JAK2 inhibitor that can induce apoptosis of human erythroleukemia cells by inhibiting the JAK2-STAT signaling.

**Electronic supplementary material:**

The online version of this article (10.1186/s13046-019-1062-x) contains supplementary material, which is available to authorized users.

## Background

The myeloproliferative neoplasms (MPN), including polycythemia vera (PV), essential thrombocythemia (ET), and primary myelofibrosis (PMF), are characterized by elevated bone marrow production of erythrocytes and megakaryocytes. This group of clonal hematopoietic malignancies is characterized by an increased risk of acute myeloid leukemia (AML) transformation [1–3]. JAK2, a member of the JAK family (JAK1, JAK2, JAK3, and Tyk2), is crucial for signal transduction downstream of the activation of erythropoietin, thrombopoietin and other related receptors which control erythrocyte and megakaryocyte production [4]. Somatic gain-of-function mutations in JAK2 are associated with myeloproliferative diseases [5]. The point V617F mutation in the JAK2 JH2 pseudokinase domain results in constitutive JAK2 kinase activity, driving cell survival and proliferation independently from signals triggered by cytokine binding [6]. This point mutation in JAK2 is strongly associated with myeloproliferative neoplasms. The JAK2^V617F^-activating mutation is highly prevalent in MPNs, with frequencies estimated at approximately ~ 95% in PV and ~ 50% in PMF and ET [7]. JAK2 mutations have also been identified in patients with acute lymphoblastic leukemia (ALL), AML, and acute megakaryoblastic leukemia (AMKL) [8]. Further studies have shown that the JAK2-STAT pathway can prevent apoptosis and promote cell growth in numerous of myeloid leukemia and solid tumors [9]. Due to the crucial role played by active JAK2 in tumor cell transformation and proliferation, as well as on the increased prevalence of the JAK2^V617F^ mutation in MPNs, JAK2 has become a potential molecular target for therapeutic intervention in MPN and other malignancies associated with abnormal JAK2-STAT signaling.

The discovery of gain-of-function mutations like V617F in the pseudokinase domain of JAK2 in MPN provided a strong rationale for the use of JAK inhibitors (Jakinibs). The potent JAK1/2 inhibitor ruxolitinib was the first FDA-approved Jakinib for the treatment of myeloproliferative diseases. The efficacy of ruxolitinib, a first-generation Jakinibs, has been convincingly demonstrated in patients with myeloproliferative diseases [10]. Although the efficacy of many first-generation Jakinibs (tofacitinib, oclacitinib, baricitinib) has been demonstrated, they non-selectively inhibit multiple JAKs and consequently inhibit a relatively broad spectrum of cytokines. The non-selectivity of first-generation Jakinibs could be the reason for their adverse effects, and the question naturally arises as to whether targeting a single JAK would show superior effects. Hence, the next-generation of Jakinibs could be designed with improved selectivity, maintaining their efficacy while improving their safety profile. Indeed, more than 25 selective JAK inhibitors are currently being investigated for various conditions, including autoimmune diseases and malignancies [11].The aim of these more selective second-generation Jakinibs, in theory, is to provide effective treatment for inflammatory or malignant diseases with reduced adverse events.

Herein, we report the identification and evaluation of ZT55, a natural product derived from *Isatis indigotica* Fort. (a popular, traditional Chinese medicinal herb), discovered by means of a high-throughput screening system and showing potential JAK2-selective inhibitory activity. The effects of ZT55 were investigated on the constitutive phosphorylation of the JAK2/STAT signaling pathway in the HEL (human erythroleukemia) cell line, carrying the homozygous JAK2^V617F^ mutation. Furthermore, we evaluated the efficacy of ZT55 in cellular and animal models of hematological malignancy, as well as its effects on primary cells derived from patients with myeloproliferative disease. We also investigated its effects on proliferation, apoptosis, and cell cycle progression. According to our in vitro and in vivo assays, ZT55 potently and selectively inhibited JAK2, but not JAK1 or JAK3. In addition, it suppressed the kinase activity of the JAK2^V617F^ protein and inhibited the phosphorylation of downstream transcription factors. ZT55 also inhibited the proliferation of HEL cells and induced apoptosis and cell cycle arrest at the G2/M phase. Moreover, we found that ZT55 suppressed the proliferation of colony-forming cells derived from human MPN patients carrying the JAK2^V617F^ mutation. This study suggests that ZT55 represents a new class of highly-selective, small-molecule therapeutic agents for the treatment of myeloproliferative neoplasms caused by the activating V617F mutation in JAK2.

## Methods

### Reagents and antibodies

ZT55 was synthesized by the Chinese Academy of Medical Sciences and Peking Union Medical College (CAMS & PUMC, Beijing, China). Anti-phospho-JAK1 (Y1022/1023), anti-JAK1, anti-phospho-JAK2 (Y1007/1008), anti-JAK2, anti-phospho-JAK3 (Tyr980/981), anti-JAK3, anti-phospho-STAT5 (Tyr694), anti-STAT5, anti-phospho-STAT3 (Tyr705), anti-STAT3, anti-Bcl-2, anti-Bax, anti-SOCS1, anti-SOCS3 and anti-GAPDH antibodies were purchased from Cell Signaling Technology (CST, Danvers, MA, USA). Recombinant human JAK1, JAK2, and JAK3 were purchased from Thermo Fisher Scientific (Waltham, Massachusetts, USA).

### Cell-free kinase activity assays

Homogeneous time-resolved fluorescence (HTRF) assays were conducted to evaluate the inhibition of JAKs by different compounds [12]. The assays were performed with the HTRF KinEASE kit (Cisbio Bioassays, Codolet, France), according to the manufacturer’s instructions. Briefly, test compounds were diluted in DMSO with a tenfold gradient series to generate a 6-point curve with an initial concentration of 10 μM. The enzymes were mixed with the test compounds and the peptide substrates in kinase reaction buffer. Following the addition of related reagents, the signal of time-resolved fluorescence energy transfer (TR-FRET) was detected using a Synergy H1 microplate reader (BioTek Instruments, Winooski, Vermont, USA). The half maximal inhibitory concentration (IC_50_) was calculated by nonlinear regression.

### Molecular docking

Molecular docking of ZT55 into the three-dimensional X-ray structures of JAK family members (JAK1, PDB code: 5WO4; JAK2, PDB code: 5UT6; JAK3, PDB code: 5TTU) was simulated using the graphical user interface DS-CDOCKER with Discovery Studio [13–15]. The protein active sites for docking were determined from the inhibitor binding sites in co-crystal structures of the protein complexes retrieved from the RCSB Protein Data Bank. Following the removal of the inhibitor, all bound waters and ligands were excluded, hydrogen atoms were added, and incomplete side chain residues were corrected. For ligand arrangement, ZT55 was constructed, minimized and prepared. Subsequently, molecular docking was executed by inserting the molecule into the binding pocket of JAKs family members based on the binding mode. At the end of the molecular docking simulations, the types of interactions occurring between the proteins and the ligand-based pharmacophore model were analyzed.

### Cell culture and proliferation assay

MCF-7, BT549, K562, KCL-22, Jurkat, Raji, U266, HEL, TF-1, NB4, and Molt-4 cells were obtained from the Chinese Academy of Sciences Cell Library and were maintained in RPMI-1640 medium with 10% heat-inactivated fetal bovine serum (Gibco, Thermo Fisher Scientific) in a humidified incubator at 37 °C and 5% CO_2_. For proliferation assays, 5000–10,000 cells/well were added in triplicate to 96-well plates and treated with increasing inhibitor concentrations. Cell viability and the IC_50_ were assessed after 48 h with the CellTiter-Glo assay MTS reagent (Promega, Madison, WI, USA) [16, 17]. The absorbance at 490 nm was measured with a Synergy H1 microplate reader. Concentration response curves were plotted to determine the IC_50_ values of the compounds. In addition, the morphology of ZT55-treated HEL cells was monitored after 24 h by means of a BioTek Cytation 5 imaging reader. The viability of HEL cells after exposure to ZT55 for 24, 48 and 72 h was also analyzed with MTS reagent to clarify the time-effect relationship.

### Flow cytometry

Apoptosis of ZT55-treated cells was assessed by FACS analysis after staining with Annexin-V and propidium iodide (PI) [18]. HEL cells (10^6^ cells/ml) were treated for 24, 48 or 72 h with different concentrations of ZT55 (0, 12.5, 25, or 50 μM). The cells were then harvested, washed twice with ice-cold PBS, and stained with Annexin-V FITC (BD Biosciences, Franklin Lakes, NJ, USA) for 15 min at room temperature. Cells were then washed with PBS to remove unbound antibody and PI (BD Biosciences) was added. Samples were analyzed with a FACS BD verse cytometer (BD Biosciences).

For cell cycle assay, HEL cells were seeded at a density of 5 × 10 ^5^ cells/ml and treated for 24 h with the serial concentration of ZT55. After treatment, cells were fixed with 70% ice-cold ethanol, stained with 20 ng/ml of PI and analyzed with a FACS BD verse cytometer [19].

To characterize expanded MPN blasts, cells were labeled with monoclonal antibodies against CD34^+^ (BD Biosciences) and analyzed with a FACS BD verse equipped with the CellQuest Pro software (BD Systems, San Jose, CA, USA) [20].

### Caspase assays

HEL cells were treated with ZT55 at concentrations ranging between 12.5 μM and 50 μM for 24 h. Caspase-3/7 and caspase-9 activities were measured using the Caspase-Glo 3/7 and Caspase-Glo 9 assays, respectively (Promega, Madison, WI, USA) [17, 21].

### Western blots

Cells were treated with ZT55 for 2 h before being lysed with Cell Lysis Buffer (Thermo Fisher Scientific), containing PMSF, complete protease inhibitors and phosphatase inhibitors (Thermo Fisher Scientific). Protein lysates were quantified by means of the BCA assay (Thermo Fisher Scientific). Similar protein amounts were mixed with loading buffer, boiled for 5 min, and separated by means of 10% SDS-polyacrylamide gel electrophoresis. Gels were blotted onto nitrocellulose membranes, followed by membrane blocking with 5% skim milk buffer, overnight incubation at 4 °C with the primary antibodies dissolved in blocking solution, and then incubation with the secondary antibodies. Immunocomplexes were visualized with the enhanced chemiluminescence detection system (ECL). The primary antibodies were directed against p-JAK1, JAK1, p-JAK2, JAK2, p-JAK3, JAK3 p-STAT3, STAT3, p-STAT5, STAT5, SOCS1, SOCS3, Bcl2, Bax and GAPDH.

Peripheral blood mononuclear cells were separated using Ficoll and then treated ex vivo with increasing concentrations of ZT55 or DMSO for 4 h. Immunoblotting was performed as previously described and blots were probed with antibodies against p-JAK2, JAK2, pSTAT3, STAT3, pSTAT5, STAT5 and GAPDH (CST, Beverly, MA, USA) [22].

### ZT55 in vivo efficacy studies

Animal studies were conducted following the protocols approved by the Experimental Animal Welfare and Ethics Committee of the Institute of Materia Medica, Chinese Academy of medical Sciences (No.001004). Female, athymic BALB/c nude mice were obtained from Weitongda Co., Ltd. (Beijing, China) and were 6–8 weeks old at the time of tumor implantation. 1 × 10^6^ cells HEL cells were resuspended in 50 μl serum-free growth medium, mixed 1:1 with Matrigel (BD Bioscience) and injected subcutaneously (total volume, 100 μl) with a 27-gauge needle in the left armpit of mice to generate the HEL leukemia model [17, 23]. Tumor-bearing mice were randomized before the initiation of treatment based on the tumor volume, and treatment was initiated when the average tumor volume was at least 100 mm^3^. ZT55 (100 mg/kg body weight) was administered orally once a day for 12 successive days. All animals were killed 4 h after the final dose on day 12 and the tumors were harvested, weighed, measured and photographed. For histopathologic analysis, tumor specimens were fixed in 4% neutral buffered paraformaldehyde solution and embedded in paraffin. Tissue sections (4 μm) were stained with H&E and apoptotic cells detected by means of the TUNEL assay.

### Colony-forming assays

Peripheral blood samples were collected after obtaining written informed consents from newly diagnosed PV and ET patients prior to starting chemotherapy and from healthy volunteers, following the Declaration of Helsinki and the Institute of Materia Medica, Chinese Academy of medical Sciences guidelines. For the analysis of JAK2 selectivity, peripheral blood mononuclear cells from MPN patients harboring the JAK2^V617F^ mutation were treated with vehicle (DMSO) or increasing concentrations of ZT55 for 2 h. Cells were then lysed with cell lysis buffer and analyzed by Western blot with antibodies against p-JAK2, JAK2, p-STAT5, STAT5, p-STAT3 and STAT3. For the hematopoietic progenitor assays, purified CD34^+^ cells were plated (1 × 10^3^ cells/dish) in methylcellulose medium (StemCell Technologies, Vancouver, British Columbia, Canada) containing cytokines (EPO, G-CSF, GM-CSF, SCF, IL-3) and in the presence of vehicle (DMSO) or ZT55 [20, 24]. Next, the culture mixtures were transferred to Petri dishes in triplicate and incubated at 37 °C in a humidified incubator with 5% CO_2_. After 14 days, colony growths derived from erythroid (BFU-E) or myeloid (CFU-G and CFU-GM) progenitor cells were scored according to standard morphological criteria [25].

### Statistics

All statistical analyses were performed with GraphPad Prism 6 software. FACS data was acquired with a BD FACSCalibur flow cytometer and analyzed with Flowjo 7.6. Results are expressed as the means ± SEM of at least 3 independent experiments. The difference between two groups of data was evaluated by Student’s paired t-test, and the difference of multiple comparisons was analyzed using one-way ANOVA, followed by an appropriate post hoc test. A *p* value < 0.05 was considered statistically significant.

## Results

### In vitro effects of ZT55

**Fig. 1:**
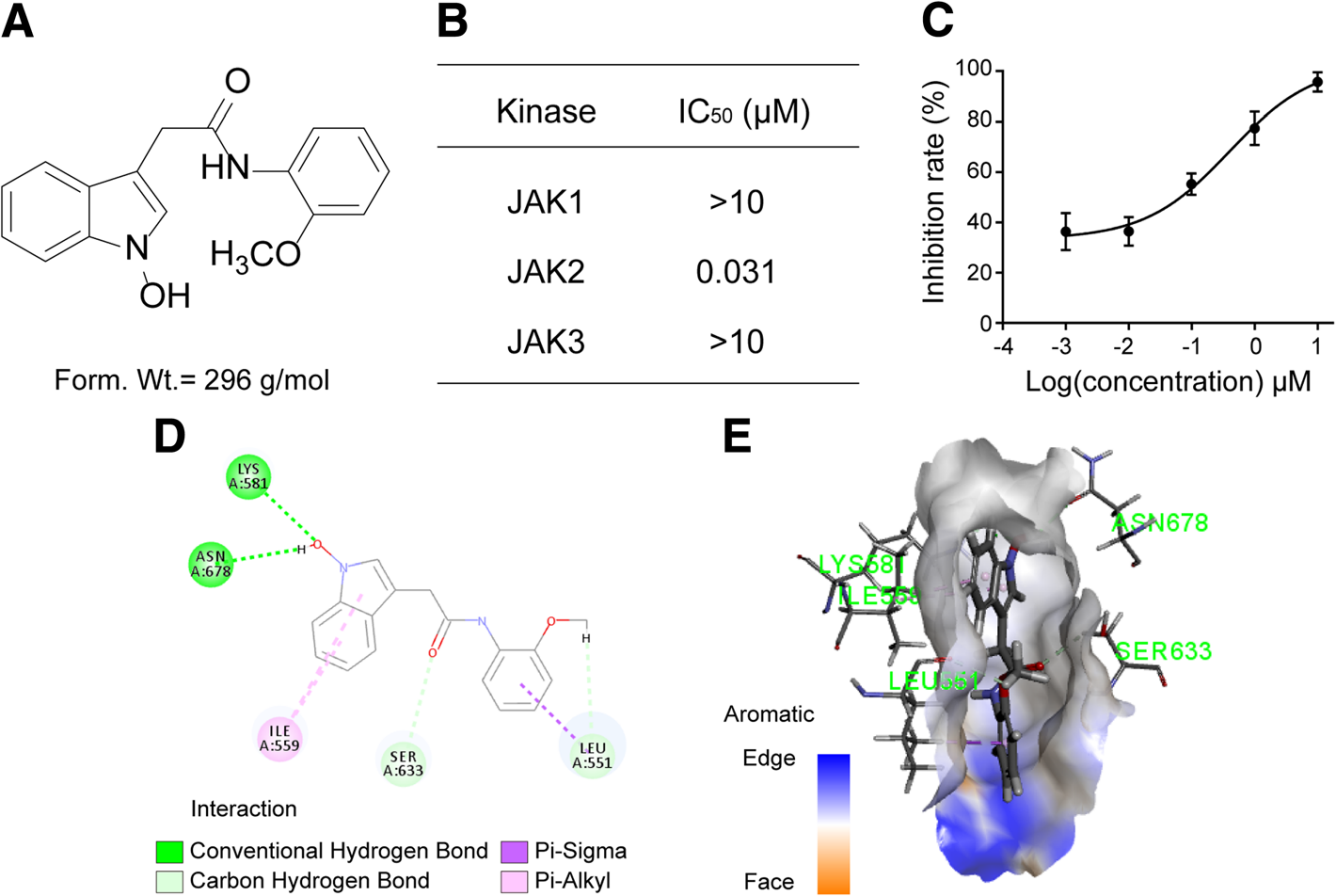
ZT55 is a biochemically selective JAK2 inhibitor. (**a**) Chemical structure and formula weight of ZT55. (**b**) The selective activity (IC_50_) of ZT55 against recombinant JAK family members using in vitro kinase assay (*n* = 6). (**c**) The curve of JAK2 kinase activity treated by ZT55 in vitro. (**d**) and (**e**) Molecular model depicting docking of ZT55 in the JAK2 ATP pocket. Key inhibitor-protein interactions, including two hydrogen bonds, three carbon hydrogen bonds, four halogen bonds, and four Pi-alkyl bonds are shown

ZT55 is a new, low molecular-weight compound derived from *Isatis indigotica* Fort. (Fig. 1a). We investigated its inhibitory effects on JAKs by means of a cell-free kinase assay with human recombinant JAK proteins. ZT55 had an IC_50_ value of 0.031 μM against JAK2 and was approximately 322-fold more selective for JAK2 than for JAK1 or JAK3 (Fig. 1b-c).

The binding affinities of ZT55 for JAK members (JAK1, JAK2, and JAK3) were predicted by virtual screening. The best docking poses of ZT55 into the JAK binding sites were evaluated with CDOCKER and the interaction energy values with JAK1, JAK2 and JAK3 were calculated to be − 42.96, − 43.93, and − 42.25 kcal/mol, respectively Additional file 1: Table S1). These results indicate that ZT55 bound to JAK2 with the lowest CDOCKER interaction energy, suggesting that ZT55 can bind more stably to JAK2 than to JAK1 or JAK3. Furthermore, the binding of ZT55 to JAK2 was compared with ruxolitinib. Compared with ruxolitinib, ZT55 showed a lower energy value for docking with JAK2 (Additional file 1: Table S2). The ZT55/JAK2 docking model in 2D and 3D (Fig. 1d-e) revealed that five amino acids (Lys-581, Asn-678, Ile-559, Ser-633, Leu-551) located in the JAK2 binding pocket played major roles in the interaction with ZT55, forming two hydrogen bonds, two carbon hydrogen bonds, one Pi-alkyl bond and one Pi-sigma bond. The molecular docking results, along with the biological assay data, suggest that ZT55 is a potential inhibitor of JAK2.

### Functional selectivity of ZT55 on JAK2-dependent cell types

**Fig. 2:**
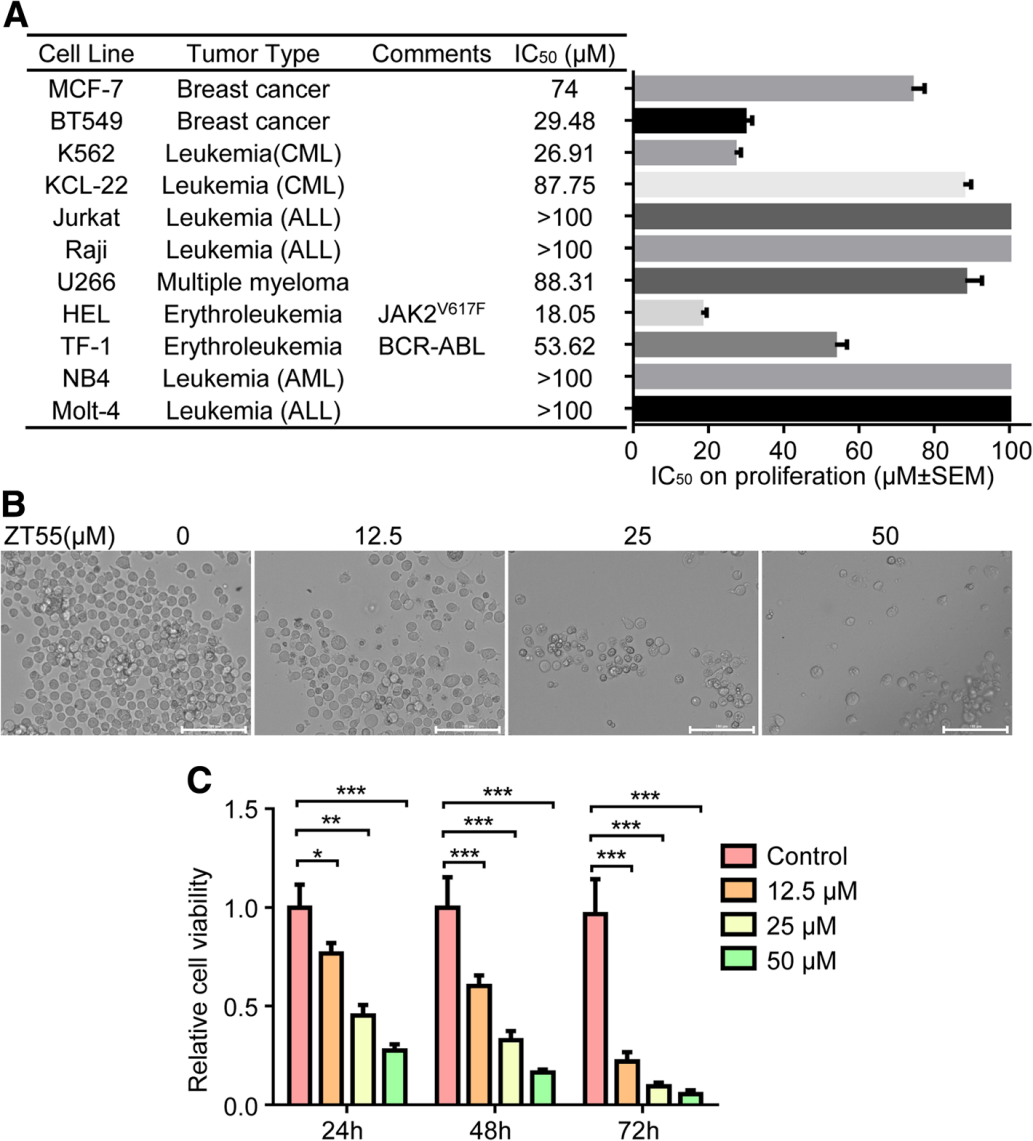
Functional selectivity of ZT55 characterized with cell proliferation and viability in cancer cells. (**a**) To determine the cell proliferation IC_50_ values, cells were treated for 48 h with various concentrations of ZT55 and their viability tested with CellTiter-Glo Assay. The mean IC_50_ results were calculated from three independent experiments. The error bars on bar graph denote mean ± SEM (*n* = 3). (**b**) Representative images were HEL cells under a phase contrast microscope after treating with ZT55 (0, 12.5, 25 or 50 μM) in 96-well plates for 24 h. Scale bar = 100 μm. (**c**) Analysis of HEL cells treated with certain concentrations of ZT55 at different time points (24, 48 and 72 h) were performed by CellTiter-Glo assay. The relative cell viability is shown as means ± SEM (*p < 0.05, **p < 0.01, ***p < 0.001)

Cell viability assay indicated that ZT55 (0–100 μM) did not show toxicity on NIH3T3 and primary T cells (See Additional file 1: Figures S1 and S2). The effect of ZT55 on tumor cell proliferation was studied in 11 human cell lines of both hematologic and solid tumor origin, including HEL cells (harboring the JAK2^V617F^ mutation), and TF-1 cells (carrying wild-type JAK2 but BCR-ABL-transformed). Interestingly, the proliferation of JAK2^V617F^ (+) HEL cells was significantly inhibited in a concentration-dependent manner after treatment with ZT55 (Fig. 2a). In fact, the cell line with the highest sensitivity towards the antiproliferative effects of ZT55 was HEL (IC_50_ = 18.05 μM). In contrast, the proliferation of BCR-ABL (+) TF-1 cell was only weakly inhibited by ZT55, and only at high concentrations. The proliferation of several solid tumor cell lines of distinct histopathological origin (MCF-7, BT549) was not affected by ZT55, consistent with the activation of other pathways, different from JAK2, in these cells. In addition, obvious changes in the morphology of HEL cells were observed after 24 h of exposure to ZT55 (Fig. 2b). The proliferation of HEL cells was analyzed with MTS. Treatment with ZT55 for 24, 48 and 72 h resulted in a concentration- and time-dependent inhibition of HEL cell proliferation (Fig. 2c).

### ZT55 induces apoptosis and cell cycle arrest of JAK2^V617F^ (+) cells at the G_2_/M phase

**Fig. 3:**
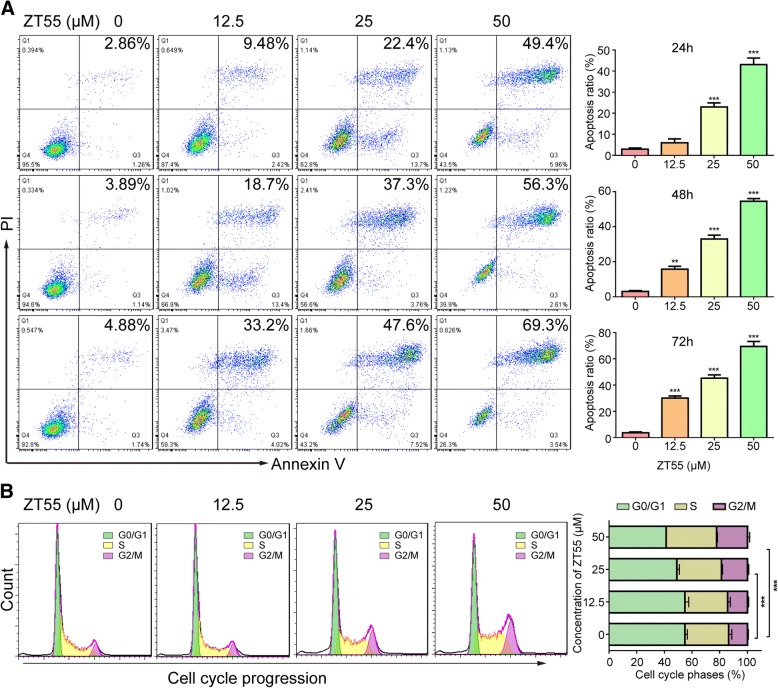
ZT55 induces cell apoptosis and cycle arrest at the G_2_/M phase in HEL cells. (**a**) Apoptosis was detected by flow cytometry in HEL cells treated with ZT55 for 24, 48 and 72 h using an annexin V/PI staining method. Representative results are shown and percentage of apoptotic cells was plotted. Data are represented as mean ± SEM (*n* = 3). **p < 0.01, ***p < 0.001 vs. control group. (**b**) HEL cells were treated with ZT55 at the concentration of 12.5, 25 or 50 μM for 24 h, and cell cycle distribution was then assessed using flow cytometry. Representative results of the cell cycle analysis and histogram of cell cycle distribution. Data are represented as mean ± SEM (*n* = 3). ***p < 0.001 vs. control group

Next, we investigated whether cellular apoptosis was being induced and the cell cycle distribution by means of annexin-V/propidium iodide (PI) double staining and flow cytometry. Compared with the control group, apoptosis was clearly induced in a time- and concentration-dependent manner in HEL cells by ZT55 (Fig. 3a). These results are consistent with the antiproliferation assays. In addition, after 24 h of exposure to ZT55, the number of HEL cells in the G_2_/M phase increased significantly in a concentration-dependent manner, accompanied by a marked decrease of cells in the G_0_/G_1_ phase (Fig. 3b). Thus, we concluded that ZT55 arrests HEL cells in the G_2_/M phase and induces apoptosis.

### Modulation of JAK2-STAT3/5 signaling pathways by ZT55

**Fig. 4:**
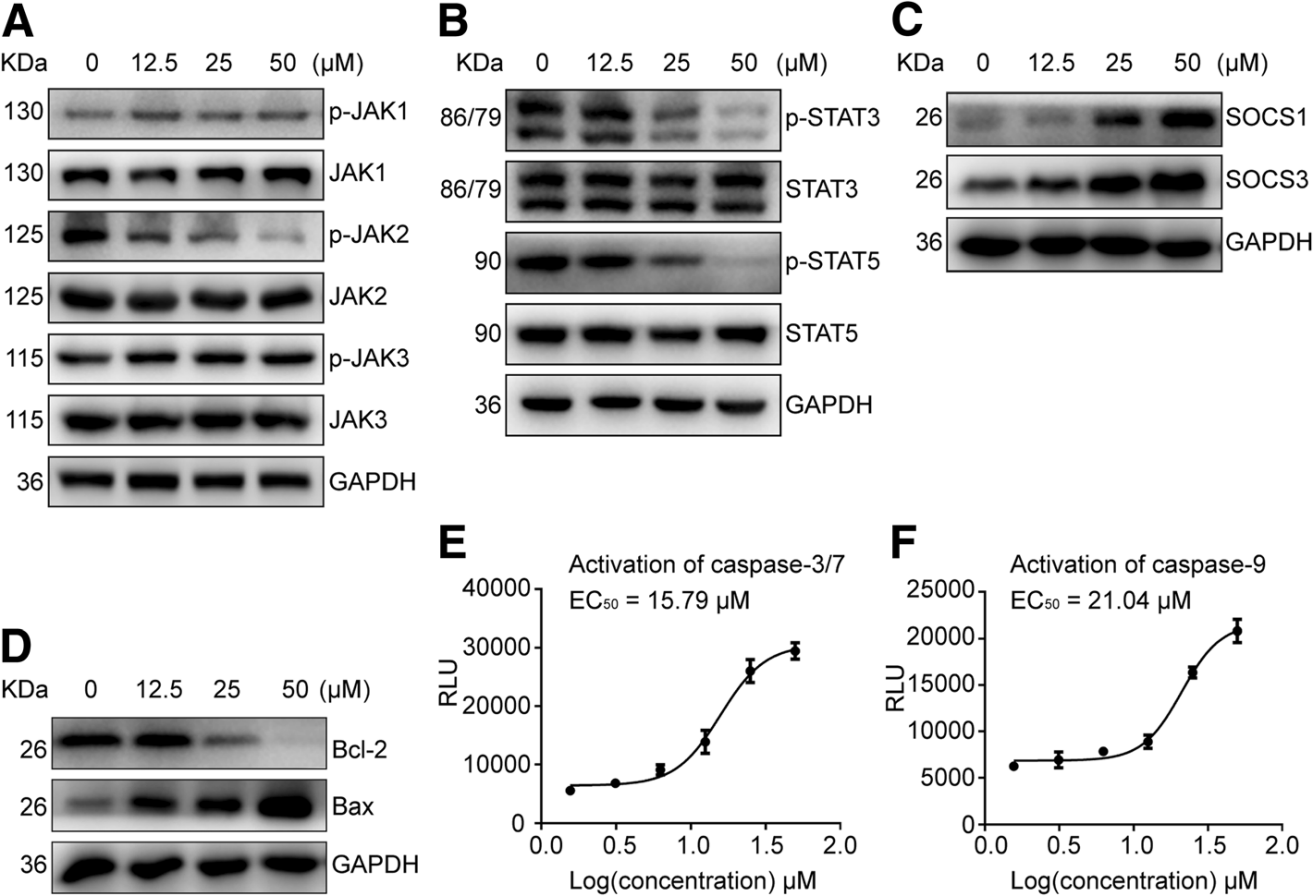
ZT55 effectively blocks JAK2/STAT3/5 signaling pathway in HEL cells. The HEL cells were treated for 24 h with the indicated concentrations of ZT55. (**a**) and (**b**) Total and phosphorylated levels of JAK1, JAK2, JAK3, STAT3, and STAT5 were determined by western blot with respective antibodies of anti-JAK1, anti-p-JAK1(y1022/1023), anti-JAK2, anti-p-JAK2(y1007/1008), anti-JAK3 and anti-p-JAK3(tyr980/981), anti-STAT3, anti-p-STAT3(tyr705), anti-STAT5, and anti-p-STAT5(tyr694). (**c**) and (**d**) The expression of SOSC1, SOSC3, Bcl2, and Bax were determined by western blot with respective antibodies of anti-SOSC1, anti-SOSC3, anti-Bcl2, and anti-Bax. (**e**) and (**f**) Caspase-3/7 and caspase-9 activity were determined by a luminescent Caspase-Glo-3/7 or Caspase-Glo-9 assays, respectively. The mean IC_50_ results were calculated from three independent experiments

To investigate whether the JAK2-inhibitory effects by ZT55 translate into modulation of JAK2-STAT signaling pathway, the phosphorylation status of JAKs and downstream proteins were determined in HEL sells. Our results indicated that ZT55 significantly inhibited the phosphorylation of JAK2, STAT3 and STAT5, but not JAK1 or JAK3 (Fig. 4a, b). In addition, the JAK2-STAT negative feedback regulatory proteins SOCS1 and SOCS3 were up-regulated by ZT55 treatment of HEL cells (Fig. 4c). To further clarify the relationship between JAK2-STAT signaling and apoptosis, related apoptotic molecules were investigated. The results showed that mitochondrial apoptotic pathway proteins of Bcl2 and Bax were regulated by JAK2-STAT signaling in ZT55-treated cells (Fig. 4d). As shown in Fig. 4e and f, there is a concentration-dependent production of cleaved forms of caspase-3/7 and caspase-9 in HELs treated with ZT55, which indicating that apoptosis associated caspases activation is triggered by ZT55. Upregulation of the pro-apoptotic protein Bax and down-regulation of the pro-survival protein Bcl2 are consistent with activation of the intrinsic pathway in apoptotic HEL cells.

### In vivo effects of 2ZT55 in a JAK2^V617F^ xenograft model

**Fig. 5:**
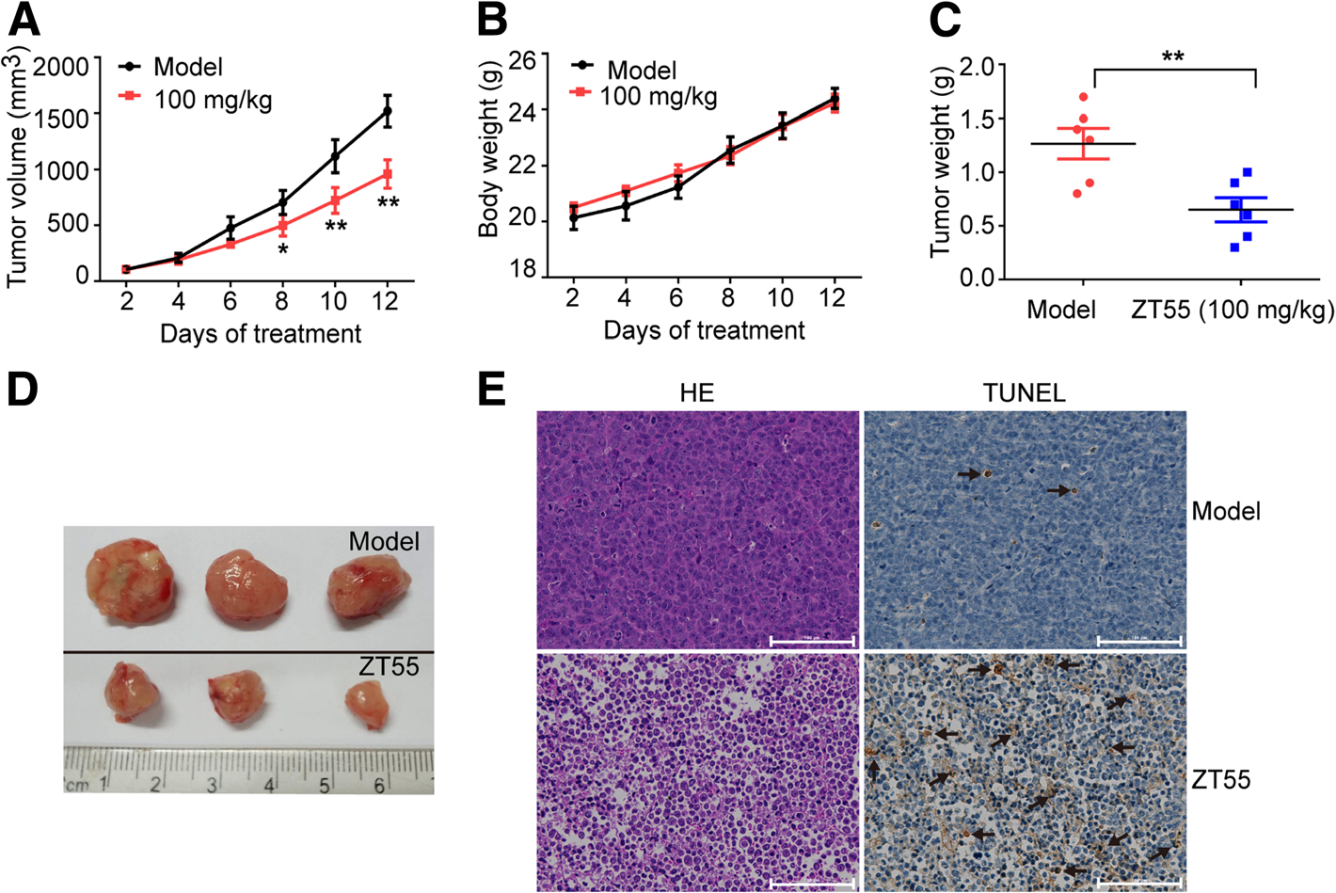
ZT55 suppresses the tumorigenicity of HEL cells in xenograft mouse model. Nude mice bearing established HEL xenograft tumors (~ 100 mm^3^) orally administered with 100 mg/kg ZT55 or vehicle control once a day for 2 weeks. (**a**) The tumor volumes and (**b**) body weight of mice were measured at the indicated time after ZT55 administration. (**c**) The tumor weights were measured to comparison. (**d**) Xenograft tumors were taken photos. (**e**) The proliferation and apoptosis of xenograft tumor cells were measured with H&E staining and TUNEL assay, respectively. Bar = 100 μm. Data are presented as mean ± SEM (*n* = 8), *p < 0.05, **p < 0.01 compared with the vehicle control group

A xenograft model was established to evaluate the antitumor efficacy of ZT55 in vivo. Specifically, HEL cells were injected into 6-week-old male BALB/c nude mice. Oral administration of ZT55 (100 mg/kg, once a day for 2 weeks) drastically attenuated the growth of subcutaneous HEL tumors in nude mice, inducing marked reductions in tumor volume when compared with control group (Fig. 5a). Interestingly, no observable weight changes were found in the ZT55 treated mice during the experimental period (Fig. 5b), suggesting that the drug did not cause systemic toxicity in vivo. The weights of ZT55-treated tumors were also significantly lower than those of vehicle-treated tumors (Fig. 5c-d). In agreement with its strong in vivo antitumor effects, ZT55 also inhibited the proliferation and enhanced apoptosis in xenograft tumors, as evidenced by HE staining and TUNEL assays performed on tumor sections (Fig. 5e).

### Effects of ZT55 on colony formation by cells derived from MPN patients

**Fig. 6:**
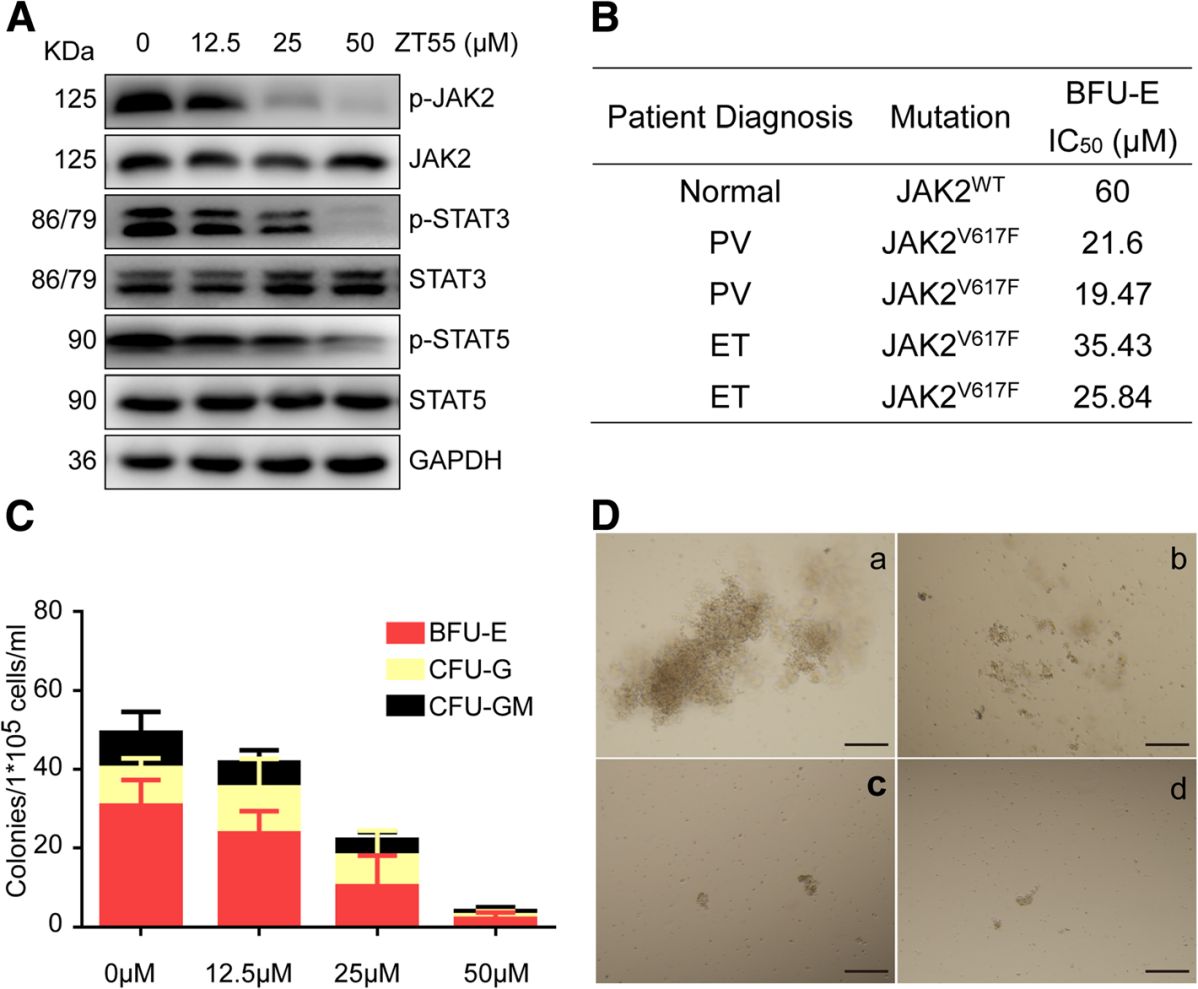
Effects of ZT55 on hematopoietic colony growth of MPN patients with activating JAK2^V617F^ mutation. (**a**) Total and phosphorylated levels of JAK2, STAT3, and STAT5 were determined by western blot in peripheral blood mononuclear cells isolated from MPN patients harboring JAK2^V617F^ mutations in the presence of indicated concentrations of ZT55. (**b**) Inhibitory efficacy (IC_50_) of ZT55 on BFU-E colony growth of cells derived from normal healthy volunteers or MPN patients harboring JAK2^V617F^ mutations was determined, each value represents each MPN patient’s BFU-E measured IC_50_ in triplicate. (**c**) Cytokine-supported colony growth of erythroid cells (BFU-E, red bars), granulocytes (CFU-G, yellow bars) or granulocyte-macrophage progenitors (CFU-GM, black bars) from MPN patients harboring JAK2^V617F^ mutations with the indicated concentrations of ZT55. Error bars indicate the SEM of mean values from three independent specimens measured in triplicate. (**d**) Representative photomicrographs of colonies derived from MPN patients’ cells plated in methylcellulose supplemented with cytokines are shown in various concentrations of ZT55 (a: 0 μM, b:12.5 μM, c:25 μM, d:50 μM), bar = 50 μm

Next, we investigated the effects of ZT55 on primary cells obtained from patients with JAK2^V617F^-driven disease. We analyzed the expression of key proteins by Western blot in peripheral blood mononuclear cells isolated from MPN patients harboring JAK2^V617F^ mutations in the presence of varying concentrations of ZT55. Our results showed a robust induction of this pathway and a concentration-dependent inhibition by ZT55, an observation that agrees with the fact that HEL cells rely mainly on the JAK2 signal transduction pathway (Fig. 6a).

In order to further evaluate the effects of ZT55 on primary JAK2^V617F^ cells in MPN patients, the colony growth assays were performed on primary hematopoietic progenitor cells isolated from the peripheral blood of ET and PV patients with the JAK2^V617F^ mutation or of health volunteers (JAK2^WT^) with cytokine supported media. In the presence of EPO, ZT55 inhibited the burst forming unit-erythroid (BFU-E) growth of clonogenic progenitors with IC50 ranging from 19.47 to 35.43 μM for MPN-patient donors and 60 μM for healthy donors, indicating an increased sensitivity to JAK2 inhibition compared with normal healthy volunteers (Fig. 6b). Under this condition, ZT55 remarkably inhibited the growth of clonogenic progenitors of erythroid (BFU-E) and myeloid origin (CFU-M/CFU-GM) with a concentration-dependent manner (Fig. 6c). In agree with this data, ZT55 had a more pronounced concentration-dependent inhibitory effect on the colony size of erythroid colonies derived from MPN patients with JAK2^V617F^ mutation than on those derived from healthy controls with JAK2^WT^ (Fig. 6d). Taken together, the data suggest that ZT55 obviously suppresses hematopoietic progenitor cell colony formation, and that the cells isolated from patients (PV and ET) carrying JAK2^V617F^ mutation are more sensitive than that of health volunteers with JAK2^WT^.

## Discussion

The cytokines that bind type I and II cytokine receptors regulate many important cellular functions such as survival, proliferation and differentiation, and are also directly implicated in the pathogenesis of numerous inflammatory and immune disorders. This important role of cytokines explains the success of ‘biologic therapies’ for the treatment of autoimmune diseases. Cytokines largely depend on the JAK/STAT signaling pathway to exert their effects. Thus, targeting JAKs with small molecules offers novel and exciting opportunities to treat inflammatory and immune disorders. Until now, almost all of the small molecule Jakinibs approved by the US Food and Drug Administration have been designed and synthesized based on the structural characteristics of JAKs. Here, we report for the first time the effects of a novel natural product derivative, ZT55, a selective JAK2 inhibitor, on cells from patients with myeloproliferative disorders. The in vitro data showed that ZT55 had higher selectivity for JAK2 than for JAK1 or JAK3. Virtual screening supported this conclusion. Furthermore, ZT55 suppressed the in vivo growth of HEL xenograft tumors and blocked erythroid as well as myeloid colony ex vivo formation by progenitor cells derived from patients carrying the JAK2^V617F^ mutation.

Constitutive JAK2 signaling is a key feature underlying the pathogenesis of myeloproliferative neoplasms. Further studies specifically identified JAK2^V617F^, a recurring gain-of-function mutation, in patients with myeloproliferative disorders [8]. Therefore, inhibition of JAK2 has become an attractive approach for the treatment of PV, ET, and PMF. Our study described a highly selective inhibitor of JAK2, ZT55. Our results using a cell-free kinase activity assay with human recombinant JAK enzymes showed that the IC_50_ of ZT55 against JAK2 was 0.031 μM. This analysis indicated that ZT55 was approximately 322-fold more selective for JAK2 than for JAK1 or JAK3. Virtual screening was also performed to predict the binding affinity of ZT55 for JAK2. These data suggests that ZT55 is more selective for JAK2 rather than for JAK1 or JAK3.

The JAK-STAT pathway is recognized as an evolutionarily conserved signaling pathway associated with the type I/II cytokine receptor family which regulates various cellular functions [26, 27]. The V617F point mutation in JAK2 results in constitutive tyrosine phosphorylation of JAK2 and promotes cell survival and proliferation independently from signals received by cytokine binding. In our study, ZT55 effectively inhibited the JAK2-STAT3/5 signaling pathway in a JAK2^V617F^-expressing cell line. Interestingly, ZT55 inhibited the phosphorylation of JAK2 at the Y1007/8 residue which constitutively activates JAK2^V617F^ in cell lines carrying this mutation, such as HEL, but it did not affect JAK1 or JAK3. Inhibition of downstream signaling events also was observed, namely suppression of phosphorylation of STAT3 and STAT5. Although aberrant activation of STAT3 or STAT5 has been reported in a variety of human cancers, determining the activation state of STAT3 or STAT5 may permit evaluating the clinical potential of JAK2 inhibitors [28]. Suppressors of cytokine-signaling (SOCS) proteins are mainly negative feedback regulators of cytokine signal transduction. SOCS1 and SOCS3, containing a kinase inhibitory region, are normally capable of binding to wild-type JAK2 and inhibiting JAK2 activity by acting as pseudosubstrates in the JAK2-STAT signaling pathway [6]. The present study indicated that ZT55 increased the expression of SOCS1 and SOCS3, which are downregulated in the constitutively activated JAK2^V617F^-HEL cell line. These results suggest that the constitutive activation of JAK2^V617F^ by the single mutation can still be regulated by SOCS proteins.

Suppression of the JAK-STAT pathway by ZT55 inhibited proliferation and induced apoptosis and cell cycle arrest in a cell line carrying the JAK2^V617F^ mutation. Cell proliferation assays indicated that ZT55 showed greater anti-proliferative effects in cell lines with activated forms of the JAK2^V617F^ mutation. The concentration- and time-dependent anti-proliferative effects of ZT55 were only observed in cells carrying activating JAK2 pathway mutations, not in ALL, AML, CML or wild type cell lines. Apoptosis or cell cycle arrest is an important inducer of inhibition of cell proliferation [29]. Our data showed that ZT55 triggered endogenous apoptosis by inhibiting Bcl-2 expression and inducing caspase-9 activation in a JAK2^V617F^-expressing cell line. Furthermore, ZT55 induced cell cycle arrest at the G2/M phase. These results imply that the growth inhibition induced by ZT55 can be explained by apoptotic induction and cell cycle arrest [30]. These findings support the notion that ZT55 selectively inhibits JAK2, preferentially modulating the JAK2^V617F^-carrying cells.

To study the preclinical efficacy of ZT55 for the treatment of myeloproliferative diseases, a nude mouse xenograft model was established by injecting animals with HEL cells expressing the JAK2^V617F^ mutation. We observed that ZT55 clearly prevented tumor growth in a dose-dependent manner. In contrast, mice weights were not significantly different between the ZT55-treated and control groups. In addition, based on microscopic observations, we found no evidence of organ dysfunction or growth disorders during the period of drug administration (data not shown). Taken together, these results support the notion that ZT55 is a potent antitumor agent against HEL cells. The in vivo data suggests this is a promising approach for the treatment of JAK2V617F-positive myeloproliferative diseases.

Another way of assessing the therapeutic effects of selective JAK2 small-molecule inhibitors targeting the V617F point mutation is by ex vivo tests using primary cells derived from MPN patients carrying the JAK2^V617F^ mutation. Uncontrolled proliferation of multipotent hematopoietic progenitor cells is a characteristic of clonal malignancies such as the MPNs [31, 32]. Therefore, primary peripheral blood mononuclear cells were collected from ET and PV patients carrying the JAK2^V617F^ mutation and from healthy volunteers to assess the growth of colony-forming cells in the presence of ZT55. Our studies showed that ZT55 blocked endogenous erythroid colony formation. These data are consistent with ongoing trials of various JAK2 inhibitors in patients with myeloproliferative disease. In addition, the levels of phosphorylation of JAK2 and downstream STAT3/5 factors in primary peripheral blood mononuclear cells from MPN patients carrying the JAK2^V617F^ mutation were also analyzed, and the results indicated significant inhibition by ZT55. Based on these data, we propose that the efficacy of ZT55 in MPN patients carrying the JAK2^V617^ point mutation could be tested in clinical trials.

## Conclusions

In summary, ZT55, a novel, small JAK2-selective inhibitory molecule showed significant anti-proliferative effects in HEL cells carrying a constitutive activating mutation in the pseudokinase domain of JAK2. ZT55 induced apoptosis and cycle arrest at the G_2_/M stage, effectively blocked tumor growth in a concentration-dependent manner and inhibited colony formation by primary peripheral blood mononuclear cells derived from patients carrying JAK2^V617F^. Collectively, these in vitro and in vivo data provide compelling arguments for a more extensive clinical evaluation of ZT55 in MPN patients with the JAK2^V617F^ mutation.

## Additional file

Additional file 1: **Table S1.** The CDOCKER-interaction energy values when the best poses of ZT55 docking with the binding sites of JAKs family. **Table S2.** The CDOCKER-interaction energy values when the best poses of ZT55 and ruxolitinib docking with the binding sites of JAK2. **Figure S1.** The cell viability of ZT55 on NIH3T3 was assessed by MTS assay. **Figure S2.** The cell viability of ZT55 on primary T cells was assessed by MTS assay. (DOCX 98 kb)

## Abbreviations

ALL: Acute lymphoblastic leukemia
AMKL: Acute megakaryoblastic leukemia
AML: Acute myeloid leukemia
FACS: Fluorescence activated cell sorting
HEL: Human erythroleukemia cell line
HTRF: Homogeneous time-resolved fluorescence
JAK: Janus kinase
MPN: Myeloproliferative neoplasms
PI: Propidium iodide
SOCS: Suppressors of cytokine-signaling
STAT: Signal transducer and activator of transcription
