# Supplementary material for: Discovery and evaluation of ZT55, a novel highly-selective tyrosine kinase inhibitor of JAK2V617F against myeloproliferative neoplasms
Source: J Exp Clin Cancer Res. 2019 Feb 4;38:49. doi: 10.1186/s13046-019-1062-x (PMC6360668; doi:10.1186/s13046-019-1062-x)
Supplement: Supplementary file 1 — Table S1. The CDOCKER-interaction energy values when the best poses of ZT55 docking with the binding sites of JAKs family. Table S2. The CDOCKER-interaction energy values when the best poses of ZT55 and ruxolitinib docking with the binding sites of JAK2. Figure S1. The cell viability of ZT55 on NIH3T3 was assessed by MTS assay. Figure S2. The cell viability of ZT55 on primary T cells was assessed by MTS assay. (DOCX 98 kb) [file 13046_2019_1062_MOESM1_ESM.docx]

**Supplementary materials**

**Tables**

Table 1. The CDOCKER-interaction energy values when the best poses of ZT55 docking with the binding sites of JAKs family.

| Enzyme | JAK2 | JAK1 | JAK3 |
| --- | --- | --- | --- |
| CDOCKER interaction energy (kcal/mol) | -43.93 | -42.96 | -42.25 |

Table 2. The CDOCKER-interaction energy values when the best poses of ZT55 and ruxolitinib docking with the binding sites of JAK2.

| Enzyme | JAK2 | |
| --- | --- | --- |
| Compound | RUX | ZT55 |
| CDOCKER interaction energy (kcal/mol) | -37.98 | -43.93 |

**Figures**


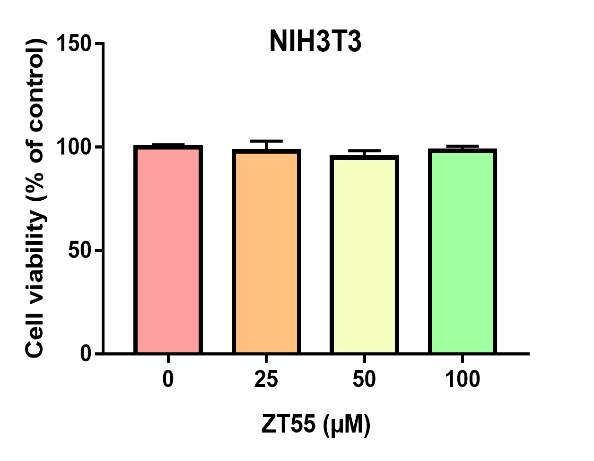


Figure 1. The cell viability of ZT55 on NIH3T3 was assessed by MTS assay. To determine the cell toxicity, NIH3T3 cells were treated for 48 h with various concentrations of ZT55 and their viability were tested by MTS Assay. The results were calculated from three independent experiments. The error bars on bar graph denote mean ± SEM (n = 3).


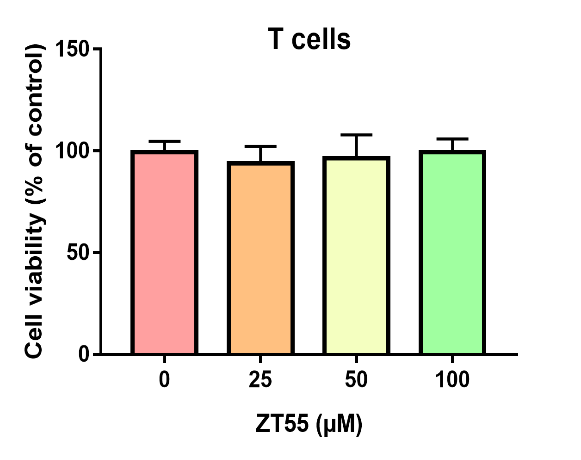


Figure 2. The cell viability of ZT55 on primary T cells was assessed by MTS assay. To determine the cell toxicity, T cells isolated from spleen of C57BL/6 mouse were treated for 48 h with various concentrations of ZT55 and their viability were tested by MTS Assay. The results were calculated from three independent experiments. The error bars on bar graph denote mean ± SEM (n = 3).
